# Dr. Bigelow’s Address

**Published:** 1865-07

**Authors:** 


DR. BIGELOW'S ADDRESS.

By direction and under the authority of the Committee of Arrangements, I bid you cordial welcome to the metropolis of New England. Sixteen years ago, these halls were honored by your distinguished presence; and since that time, although a bloody war has devastated the land, twice have you met together, under the sheltering protection of cities far distant from each other, to lay an offering upon the altar of science. We are now once again assembled, to unite in a single common sentiment of congratulation at the advent of peace, for which we have so long watched, and in whose genial sunshine the flowers of science may again expand.

The American Medical Association was at no remote period one of the prosperous institutions of the country. From a small and perhaps doubtful beginning, it had risen by a continuous and steady growth to become one of the successful enterprises of the age. The great cities of the North hailed its advent with acclamation, while further South, the hospitable homes of Richmond and Charleston, of Nashville and St. Louis, were thrown open for its warm and generous reception.

Such was our enviable position four years ago, when the demon of insurrectionary strife exhaled its poisonous and blasting breath over the fairest and most fertile portion of our common country. Cut off from intercourse with their Northern brethren, deprived of the opportunity of attaining light and truth, borne down by relentless exactions and unmerciful conscription, by devastating war and its inevitable sequels of poverty and bereavement, our Southern States have been to us for four years an alien and a hostile land.

During this long and weary period, the steadfast edifice of the Republic has breasted the battle and the storm; an insidious and fatal bolt at last descending to rive its highest pinnacle. We recover from the shock to thank God, that as the tumult ceases, and the smoke clears away, the grand old edifice still lifts its head among the nations, unshaken in its foundations, untarnished in its glory, an impregnable tower of strength, and the hallowed shrine of patriotism. The Southern States have at last succumbed before the persevering valor and terrible unanimity of the North, and the jarring elements have at last found repose, after the convulsions of a great transition period, not in the replacement of the old strata, but by the gravitation of the social system into the final and inevitable equilibrium of human rights.

While the whole country is occupied with the great and difficult problem of reconstructing the Union, that we may come out of this ordeal a wiser, a more stable, and a more prosperous people, it is for us to consider whether we cannot do something to render our own Association a more efficient and a more productive institution. No one can doubt that the medical science of this country, now ostensibly represented in this body, is destined one day to occupy a very high place in the medical history of the world. The American mind, the practical ability of which no one has ever doubted, is devoting itself more and more to the study, by exact experimental observation, of abstract truth, each year augmenting the number of medical philosophers devoted to scientific research at the sacrifice of professional and personal interest. It requires no prophet to foretell that they will identify this Association with illustrious labors whose magnitude and importance will henceforth keep pace with the invigorated growth of the Republic.

And who shall set bound or limit to the vast future of this country? If we believe with the great philosopher that, "in the youth of a State arms do flourish; in the middle age of a State, learning." Who that has witnessed the Titanic conflict between opposing hosts, such as the history of the world has rarely seen confronted, who that contemplates the magnitude of this broad continent, its millions of acres of waving grain, the treasures buried in its bosom, the iron, the gold and silver, and coal and oil more precious than these; the great liquid highways that bind together the North and South and East and West in one indissoluble bond of natural union; the energy of its people, the enormous territory now first thrown open to the intelligent efforts of free labor, who can fail to discern in these combining elements of prosperity the stalwart youth of a colossal nation? Let your imagination for a moment contemplate the vision of its maturity and manhood; when the great Western Valley shall become a central home of letters and the arts, when the culminating light of science shall there shed its full effulgence, and the youthful giant of the Western hemisphere in his ripened strength and intellect shall challenge the place left vacant for him on our planet.

While the great Republic is accomplishing its political destiny, let us fail not to carry forward our corresponding mission of relieving human suffering, averting human disaster, and retarding human decay; not by deceptive assumptions, not by fallacious assurances, not by the dogmas which professional pride has set up, but by an earnest, impartial, and discriminating pursuit of truth, and by an unwearied effort to divorce popular error from the companionship of legitimate science. It is our duty to lay here in solid labor the foundations of an association which for a century to come shall gather to a focus and radiate the light emanating from the best minds in our profession.

We rejoice to offer an earnest expression of our gratitude to those of our medical brethren, some of whom we are proud to see among us, who have stood so nobly at their posts by sea and land, amid the carnage and the pestilence; and who, surrounded by the distractions of the camp and of the fight, have borne a conspicuous part, both by their active services and their literary labors, in upholding the honor and dignity of their profession.

Not less happy are we to see among us our brethren of those neighboring provinces, from which we have received such kindly tokens in our recent hour of national affliction. The people of this continent have so many common interests and common sympathies, that no political landmarks can render them alien to each other.

I welcome you, gentlemen, in behalf of the Committee I have the honor to represent; of the old Massachusetts Medical Society, who cherishes a matronly regard towards her younger sisters of other States; in behalf of the City of Boston, which extends to you her civic hospitality. Welcome, friends and brothers! assembled from distant regions of our common land—from the great commercial emporium through whose aortic thoroughfare pours the ceaseless tide of nations, or from the city whose traditional brotherly love echoes so freshly from the lips of all our wounded soldiers; you, brothers of New England, born to the common heritage of toil and freedom; you, whose home is by the great Western watercourses, whose blood sprang from the same fountain as our own, and has so often mingled with it again upon the battle field; and you, few we may fear, but thrice welcome, loyal and faithful brothers of the South, who have passed through the long night of trial that you might hail to-day the glorious dawn of liberty. Welcome, fellow-citizens of the redeemed Republic, whose wounds you have bound up in binding up those of her defenders. Welcome, all who honor us by their presence on this auspicious morning, which beholds the sacred emblem of liberty restored to its rightful places, tattered with bullets, stained with blood, fringed with the sable sign of mourning, but spread over every stronghold from which treason had struck it down, and soon to rekindle all its ancient glories.

At the close of Dr. Bigelow's address, the roll of registered members was read by the Secretary.

It was voted that names of persons desiring election as pernent members, on presenting the proper credentials, be handed to the Committee of Arrangements, to be hereafter reported upon.

Communications from absent members were received.

The President then proceeded to deliver the annual address, as follows, which was received with marked approbation, and at its close the thanks of the Association were voted to Dr. Davis, and a copy of it was requested for publication:—

DR. DAVIS' ADDRESS.

